# What (If Anything) is Wrong with High-Frequency Trading?

**DOI:** 10.1007/s10551-022-05145-7

**Published:** 2022-05-26

**Authors:** Carl David Mildenberger

**Affiliations:** Department of Philosophy, University of Zurich, Zollikerstrasse 117, 8008 Zurich, Switzerland

**Keywords:** Low-latency trading, Arbitrage, Information asymmetry, Systemic risk, Speed

## Abstract

This essay examines three potential arguments against high-frequency trading and offers a qualified critique of the practice. In concrete terms, it examines a variant of high-frequency trading that is all about speed—low-latency trading—in light of moral issues surrounding arbitrage, information asymmetries, and systemic risk. The essay focuses on low-latency trading and the role of speed because it also aims to show that the commonly made assumption that speed in financial markets is morally neutral is wrong. For instance, speed is a necessary condition for low-latency trading’s potential to cause harm in “flash crashes.” On the other hand, it also plays a crucial role in a Lockean defense against low-latency trading being wasteful developed in this essay. Finally, this essay discusses the implications of these findings for related high-frequency trading techniques like futures arbitrage or latency arbitrage—as well as for an argument as to why quote stuffing is wrong. Overall, the qualifications offered in this essay act as a counterbalance to overblown claims about trading at high speeds being wrong.

## Introduction

In 2010, the first “flash crash” on the financial markets took place. The *Dow Jones Industrial Average* fell about 1000 points (9%)—its second biggest intraday point decline up to that point—in mere minutes. Everybody was quick to blame the then new practice of *high-frequency trading* (HFT) for this event. But in the first paper engaging with HFT from a moral perspective, Angel and McCabe conclude that “[i]t is not the speed of the tool that matters for fairness, but what is done with it” (2013, 585).

Angel and McCabe argue that trading techniques that have always been wrong, stay wrong, irrespective of the speed with which they are executed. On the other hand, Angel and McCabe argue that, if a trading technique is morally unsuspicious if done at a low speed, so it is if done at high speed.

The tone thus set by Angel and McCabe, i.e., that speed is morally neutral, since has not been questioned. The few articles and the court cases engaging with HFT have focused on specific trading techniques often employed by high-frequency traders—such as *front running*, *quote stuffing*, *spoofing*, or order discovery and triggering strategies—rather than the practice of trading at high speeds as such (e.g. Aldridge & Krawciw, 2017; Cooper et al., 2016; Diaz & Theodoulidis, 2012; Holzer & Philbin, 2010; Martens, 2014; McLeod, 2013).

This essay puts forward a qualified critique of HFT, highlighting that speed may influence its permissibility in different ways. That is, I argue that speed is not morally neutral. Because speed shall be the issue, this essay focuses on a specific variant of HFT known as *low-latency trading* (LLT). This variant is purely about being faster than others, and thus arguably the “plain vanilla” form of HFT. Roughly speaking, a low-latency trader catches public information that (i) someone wants to buy a certain stock and that (ii) this stock is for sale on some exchange at a certain price. This trader then (very quickly) buys the stock from the seller and sells it to the buyer at a slightly higher price, making a profit.

Conceptually speaking, LLT comes down to practicing arbitrage with respect to time on the basis of an information asymmetry. This is why, after a more detailed presentation of how LLT works (section “Low-Latency Trading”), I examine the *arbitrage* (section “Hayek on the Social Usefulness of Arbitrage”) and *information asymmetries* (section “Information Asymmetries and Justice in Exchange”) at the center of LLT from a moral perspective. I engage with general arguments by Hayek (1945) on the social usefulness of arbitrage and Steiner (2019) on the legitimacy of information asymmetries. What I find is that the speed with which LLT proceeds (and the costs for establishing and profiting from it) seems to have the potential to turn LLT, all things considered, into a socially wasteful practice. This social wastefulness critique of LLT is qualified in section “Social Wastefulness, Wrongness, and Speed”. I argue how, on liberal grounds and relying on a Lockean account of social wastefulness, the traders’ being fast might be seen as what saves LLT from being wasteful (and *pro tanto* wrong).

In section “Systemic Risk”, I address the *systemic risk* created by trading at high speeds. For one might worry that, while individual acts of low-latency trading might be morally unproblematic, the practice as a whole endangers the stability of the financial system. However, I find that it is by no means easy to make a convincing analytical case that LLT is related to systemic risk (let alone that individual traders are to blame)—or that, empirically speaking, the threat of harm is substantial.

Section “Implications for Other HFT-Techniques” examines the implications for what has been found with respect to LLT for related high-frequency trading techniques. For instance, I find that there is a speed-related reason to argue that quote stuffing, while not currently deemed illegal, may be considered wrong. Section “Conclusion” offers a general conclusion.

## Low-Latency Trading

LLT is a trading technique characterized by the speed with which transactions are made, by the high frequency of transactions, and by the fact that with each transaction there only is a very small profit made. There is no official data concerning the overall volume or market share of LLT. For HFT in general, there are estimates that around 40% of all trading volume and 70% of all orders on financial markets can be traced back to it (Aldridge & Krawciw, 2017; Moser & Wunderer, 2020).

To engage in LLT, two things are needed: first, a very fast data connection; second, a software-based trading algorithm. The very fast data connection is needed so as to have access to real-time financial information and to be able to act on it before other people can. Currently, the fastest data connection between the exchanges in New York and Chicago takes around 8 ms (Moser & Wunderer, 2020). That is only about 10 microseconds above light speed.

The software-based trading algorithm is needed because executing orders at these speeds is beyond human capabilities. Just as there is a race among engineers for providing the fastest data connection, there is a race among programmers to write the fastest code.

Here is a simplified example of an instance of LLT. A big investor, pension fund *P*, decides to buy 500,000 shares of company *C*. The current share price is $19.83. Because of the size of the order and legal requirements, the pension fund sends out the buy order to several exchanges (call these *E*, *F*, *G*, and *H*) at once. Once the buy order reaches the servers of the first exchange (exchange *E*), the algorithm of low-latency trader *T* processes this input in fractions of seconds. In fact, LLT-servers typically are located right next to the servers of the exchange (so called *co-location*). This shortens the time it takes for the data to travel from the exchange’s server to the server of *T*.

Importantly, as soon as *P*’s buy order reaches exchange *E*, the buy order becomes public knowledge. That is to say, LLT is not a case of *insider trading* (cf. e.g. Engelen & Van Liedekerke, 2007; Lippke, 1993; Martin & Peterson, 1991; Werhane, 1989). Sure, *T* knows first as he invested a considerable amount of money into knowing first, and thus is able to make use of this information faster than others. But this does not change the fact that the piece of information LLT relies on to generate profits is, in principle, a public piece of information. Anybody to whom this information is worth much is free to set up their own LLT operation.

In the next step, *T*’s trading algorithm sends out its own buy order for shares of company *C* at $19.83 to exchanges *F*, *G*, and *H*. Because of the very fast data connection *T* has to these other exchanges, the algorithm’s buy order (which was made *after* the pension fund’s buy order) actually reaches the exchanges *F*, *G* and *H before* the pension fund’s order does. That is to say that LLT relies on speed both at the “information reception” level and at the “exchange” level. As a result, *T* buys up all shares of *C* at exchanges *F*, *G*, and *H* at $19.83. He then immediately sells them back to the pension fund at $19.84. For in the meantime, the pension fund’s order has arrived, but all shares at $19.83 are gone. So a higher price has to be accepted.

If *T* was able to buy, say, 300′000 shares at $19.83 at exchanges *F*, *G*, and *H* and immediately sold them back to *P* for $19.84, his profit amounts to 300,000 times $0.01, i.e., $3000. That might not seem particularly much. But it was realized in fractions of seconds. And if trading volume is sufficiently high, the small amounts add up.

From a conceptual standpoint, LLT is not new. Basically, what we are dealing with is traders practicing arbitrage with respect to time (buying at a lower price now to sell at a higher price later). They do so based on an information asymmetry. For at a certain point in time, the low-latency traders know things others do not. The idea to make money this way is as old as Thales of Miletus. As Aristotle tells us

when … [Thales] realized from his astronomy that there would be a harvest of olives, then while it was still winter and he had a little money to spare, he handed round deposits on all the oil-presses in Miletus and Chios, hiring them for a small sum, as no one was outbidding him. When the time came, and many people suddenly and simultaneously sought them, he hired them out on what terms he wished. He raked in a lot of money … . (Aristotle (1984) *Politics*, 1259a7-16)

Thales certainly was no low-latency trader, but he practiced arbitrage with respect to time based on an information asymmetry. In the next two sections, I shall critically examine these two conceptual pillars on which LLT fundamentally rests from a moral perspective.

## Hayek on the Social Usefulness of Arbitrage

In *The Use of Knowledge in Society*, Hayek stresses the importance of that kind of knowledge which is “knowledge of the particular circumstances of time and place” (1945, 521). He argues in support of the dispersed knowledge that “the man on the spot” (1945, 524) possesses—and against what he sees as an unduly fixation on the centralized, scientific knowledge experts possess and the planning they do. This is because we should not forget, as it were, who actually keeps things running in the economy.

Knowledge of the particular circumstances of time and place is what underlies arbitrage. Consequently, Hayek includes “the *arbitrageur* who gains from local differences of commodity prices” (1945, 522) in his list of socially useful people. Like somebody knowing of a machine currently underemployed, the arbitrageur possesses socially useful knowledge. And him practicing arbitrage means to put this socially useful knowledge to work for society.

According to Hayek, the arbitrageur’s business model, to buy at low prices in one location to sell at higher prices somewhere else, effectively *connects* the demand and supply at two otherwise separate locations. The arbitrageur thus increases the productive efficiency of the overall economic system—notably via having an effect on the overlapping system of prices. In line with this, Hayek attacks those who look at the arbitrageur with contempt and who think he acts disreputably or even dishonest (1945, 522).

Time is of the essence for Hayek. He emphasizes that economic change is constant and rapid—and speaks of opportunities of “fleeting moments” (1945, 522) that arbitrageurs seize. Also, he explicitly tells us that the price system fulfills its function “less perfectly as prices grow more rigid” (1945, 526), i.e., less constantly updated. Thus, while Hayek does not explicitly demand that arbitrageurs be as fast as possible, it is hard to see why he would be opposed to arbitrage processes happening ever faster.

### Hayekian Arbitrage and LLT

Although Hayek argues for the social usefulness of arbitrage in general, we may reasonably doubt whether his argument likewise applies to LLT. This is because there are noteworthy differences between the arbitrage Hayek describes and the special kind of arbitrage present in LLT.

Consider that what seems to lie at the heart of LLT is pure time arbitrage. Hayek’s arbitrageur connects different physical locations rather than simply buying at a lower price now to sell at a higher price later, without moving anything.

To be sure, one might reasonably question whether LLT actually is a form of pure time arbitrage. For low-latency traders are connecting different exchanges with their fast data lines. They transfer the information acquired at exchange *E* to exchanges *F*, *G*, and *H*. And they place buy orders at these physically distant locations based on their knowledge acquired at *E*. Thus, although LLT does not really move things other than data, there is some aspect of connecting people across space present in LLT. By comparison, Thales’ investing practices do not feature this spatial aspect. The olive presses stay right where they are.

Furthermore, even if we classified LLT as pure time arbitrage, we might ask why, if judged against the background of Hayek’s idea of desirable connection by arbitrage, there should be a morally relevant difference between connecting people across space and across time. Connecting different points in time clearly is a very useful thing from an economic perspective. Think, for instance, of how economists judge money’s ability to act as a store of value—i.e., to provide stability across time—as a crucial feature of every well-functioning currency (Mishkin, 2013, chap. 1).

But we can push this point somewhat harder. For one might ask whether LLT really connects anybody across time. LLT is all about establishing a connection faster that would have been established anyway. If we counterfactually asked: “Would the exchanges *E*, *F*, *G*, and *H* be connected anyway without low-latency traders entering the scene?”, the answer would be yes. The large buy order of *P* over 500,000 shares of *C* alone is enough to connect them, for *P* sends out this order to all exchanges. And even without LLT taking place, *P* is unlikely to pay the exact same price for all of the 500,000 shares. Larger orders take time to be effected on the market. And once all shares for $19.83 are gone, the price moves up to $19.84, $19.85, and so on for the remaining shares. To paraphrase this situation, we might say that LLT leads to *faster* accurate pricing, but not to more accurate pricing as such.^1^

In light of this, we might qualify the connecting done by low-latency traders as superfluous. Hayek’s arbitrageur makes the market more connected and contributes to more accurate pricing where LLT does not. A similar criticism would apply, it seems, to Thales’ pure time arbitrage. Eventually, prices for the use of olive oil presses would have risen anyway. Just like LLT, Thales’ arbitrage only makes prices adapt faster.

But there are two important differences between Thales’ arbitrage and LLT. First, low-latency traders are bridging much shorter periods of time. In this respect, their contribution seems even smaller than Thales’.

Second, Thales—unlike low-latency traders—runs the risk of being wrong. If he had bought the rights to use olive oil presses in future, and his prediction had turned out wrong, he actually would have lost money. Low-latency traders do not face this risk of wrongly predicting a price increase. They already know that *P*’s buy order for 500,000 shares of *C* will increase the price. In this respect, they practice “risk-free” arbitrage.^2^

This second difference forms a solid basis for arguing, with Hayek, in favor of pure time arbitrage—while not at the same arguing in favor of LLT. The idea is the following. Arbitrageurs who run the risk of being proven wrong about their predicted future price increase add a new connection across time. By adding information about the future risk of price changes to the current price of the right to use olive oil presses, Thales adds to the accuracy of the price for olive oil presses and genuinely connects two points in time. By contrast, low-latency traders just update the price faster.

To conclude, if low-latency traders do not really connect people across time, Hayek’s argument for the social usefulness of (pure time) arbitrage does not also speak in favor of LLT. This does not make arbitrage as such impermissible—or LLT outright wrong. But it is important to acknowledge that we cannot say much in favor of LLT based on a general moral theory defending arbitrage like Hayek’s.

Although speed is a necessary component for arbitrage, socially useful arbitrage has to be about more than just a little bit more speed. Especially since there is strong evidence that the arbitrage-benefits associated with LLT stand in stark contrast to its costs. The informational gains provided by LLT are minimal. Given that it takes lots of resources (building data connections across mountains, drawing in talented people, tying up capital, etc.) to set up an LLT operation, this might be seen to give us good reason to judge LLT as socially wasteful rather than useful, all things considered.

To be sure, just how much LLT actually contributes to liquid markets—or what more liquid markets are worth to a society in monetary terms—is an empirical debate that cannot be settled here (e.g. Jovanovic & Menkveld, 2016; Litzenberger et al., 2012). Neither should we jump to the conclusion that every socially wasteful practice necessarily is also wrong. But at the very least, the burden of proof that LLT is not a socially wasteful instance of arbitrage shifts to the low-latency traders.

## Information Asymmetries and Justice in Exchange

In the case of LLT, one party to the exchange (namely, *T*) knows more about the trade to be effected than the other party (namely, those who sell the shares of *C* to him). *T* knows more as he knows about the pension fund’s buy order before the sellers do. It is a question of *justice in exchange* (e.g. Cohen, 1995; Mildenberger, 2020; Nozick, 1977; Steiner, 1994) whether making a profit based on knowing something that others do not is wrong, as the exchange is to be considered unjust.

The general case of an information asymmetry is that between a seller and a buyer, with one knowing more than the other. Say the buyer knows material fact *p* about the good *g* to be exchanged—but the seller in addition knows material fact *q* about *g*. Because one party knows *p* while the other knows *p* and *q*, there is a characteristic difference in information levels.

The information asymmetry that characterizes LLT is special in that, first, LLT is about knowing things slightly earlier than others. Second, we are dealing with the somewhat untypical scenario of the buyer knowing more than the seller. Kronman (1980) presents another such case, in which a buyer rents an airplane to fly over the seller’s land—thereby detecting some geological information unknown to the seller, which makes the land more valuable. But neither characteristic of LLT is so special that it would render general moral considerations about the legitimacy of information asymmetries in exchange unsuitable to clarify the case at hand.

The question of whether information asymmetries undermine the legitimacy of exchange has recently received a systematic treatment by Steiner (2019). Steiner holds that there is a moral presupposition in favor of full disclosure, as informed choices are better choices. However, he thinks that as soon as we take into account the *costs* sometimes related to acquiring information—for the individuals acquiring the information and for society—the position that *every* information asymmetry should be resolved becomes implausible.

### Coming to Know More than Others

For Steiner, in order to judge the legitimacy of profiting based on information asymmetries, we have to answer the question how those in the know *came to know more*. Steiner stresses that requiring all information asymmetries to be resolved would take most of the incentive to discover information away (cf. Collins, 1992, 556). This could lead, in the end, to a society not tapping its full potential. For instance, there would be no incentive to even start a discovery flight for the prospective buyer of the piece of land—which might lead to the resources slumbering beneath the ground indefinitely.

A generalized duty to disclose all information would not simply be better, in the sense of constituting a Pareto-improvement (Steiner, 2019, 98). For we would have to ask: better for whom? Those who made the initial investments in acquiring information would most likely be worse off. They would be less able to strike favorable deals (that compensate them for the investment in discovering additional information they made), if they had to share all their acquired knowledge.

On the other hand, we should not ignore the costs of socially wasteful information discovery. Eisenberg, for one, points out that “requiring [full] disclosure can save the socially wasteful costs of searching for information that the other party already has or of making duplicate searches to generate the same information” (2003, 1647). If, all things considered, the costs of the prospective buyer’s flyover outweighed the informational advantage gained by this, this seems to be another instance of socially wasteful information discovery. For example, in the case of a rather small amount of mineral resources discovered, the prospective buyer’s potential profit and society’s access to new resources might be outweighed by the costs for fuel and the damage done to the climate.

Steiner’s considerations about information asymmetries thus mirror Hayek’s thoughts about arbitrage to a certain extent. Acquiring an informational advantage to profit from it often is a socially useful practice. It enlarges the pool of a society’s knowledge. But if Steiner argues that the costs of acquiring informational advantages matter in justifying less than full disclosure, it seems plausible to argue, along Steinerian lines, that the legitimacy of information asymmetries also suffers if the costs for acquiring them are too high.

In the end, if we follow Steiner, we must decide for each practice involving an information asymmetry on a case-by-case basis whether it is socially wasteful. As regards LLT, it seems a *prima facie* reasonable position to stress that the few split-seconds gained in terms of faster accurate pricing do not significantly enlarge the social pool of knowledge—while the costs for acquiring them are high.

### Continuing to Know More than Others

Further developing Steiner’s thoughts, one might argue that for deciding whether an information asymmetry undermines the legitimacy of an exchange, it does not only matter *how one comes to know more*. It also matters *why one continues to know more*, i.e., the intention of the party having an informational advantage (cf. Mildenberger, 2020, 37–38). At least three cases need to be distinguished, of which two seem morally unproblematic.

First, the better-informed party may not share the additional information with the less well-informed party *inadvertently*. A concrete example might involve a piece of information about the good to be exchanged that is not material to the seller, nor to the “standard” buyer of the good, but which for some reason matters a lot to the particular buyer in question. In such a scenario, the seller simply does not know that the piece of information is material to the buyer, and blamelessly does not share it, although in principle he would be willing to.

Second, the party with more information might be *unable to convey* the additional information to the less well-informed party. This is the case Akerlof (1970) describes in his seminal paper about the market for “lemons.” The sellers of high-quality used cars have little to no way to convey the information that they gratuitously acquired—namely, that their cars are immaculate—to potential buyers. Making that information public would be beneficial to both parties. The sellers could realize a higher price and the buyers could be sure not to buy a “lemon.” Alas, it is virtually impossible to overcome the information asymmetry completely.

Only the third case of why one party continues to know more might be morally problematic: if one party *intentionally* does not inform the less well-informed party. Notably, this is because worries of deception start to play a role. If, say, the buyer intentionally causes the seller to have a false belief that is known or believed to be false by the buyer *by means of non-disclosure* (and where there are duties to disclose), then we are dealing with deception (Mildenberger, 2020, 35–37). Although not all deception proceeds by means of non-disclosure, intentionally upheld information asymmetries are a necessary condition for deception (Steiner, 2019).

While LLT is an instance of an intentionally upheld information asymmetry, it is not an instance of deception. At no point do low-latency traders intend to cause a false belief in those wanting to sell shares of *C* at $19.83. Considerations of timing are the most relevant in supporting this point.

At the time *T* is buying from the sellers, i.e., when he knows about *P*’s buy order but the sellers do not, the sellers’ sell orders have already been registered with the exchanges *F*, *G*, and *H*. In fact, the sellers’ sell orders have been placed even before the information asymmetry has arisen, i.e., before *P* placed its buy order. At that time, the sellers have made an offer to sell a certain number of *C*’s shares at $19.83 to whoever wants to buy them. And this is just what *T* starts wanting to do as he hears from *P*’s buy order. It is not even clear that the sellers would rather have sold their shares at $19.84 or $19.85 had they known of *P*’s buy order earlier. This depends on whether the sellers’ aims are to get rid of their position of *C*’s shares as fast as possible, or if they are willing to wait a bit longer to get a better price. In any case, as the sellers place their orders before *T* does, the latter cannot cause a false belief in them with respect to their sell orders.

Also, *T* does not deceive the sellers about the nature of the good exchanged. Since low-latency traders buy and sell standardized financial products—and since one of *C*’s shares is exactly like the other—for once the mainstream microeconomic assumption that on markets *homogenous* goods are traded holds true. There is strictly no information asymmetry with respect to what is exchanged.

If LLT is not an instance of deception—and does not involve an information asymmetry about the nature of the good exchanged—what is the additional information low-latency traders possess about? It is additional information about the market conditions, i.e., which buy and sell orders there are at any given point in time. The fact that low-latency traders know the market better might spark the worry that LLT, if not deception, is a case of *insider trading* (cf. e.g. Engelen & Van Liedekerke, 2007; Lippke, 1993; Martin & Peterson, 1991; Werhane, 1989). But as discussed in section “Low-Latency Trading,” this is not the case either. LLT does not rely on possessing *private* information, but on processing *public* information faster than others.^3^

One might still object that low-latency traders’ intentionally upholding an information asymmetry is wrong for yet other reasons. In that case, one would have to come up with a convincing argument for why it is wrong to non-deceptively profit from information that is, in principle, publicly available, but that one holds faster than others. One route to try might be to come up with a non-arbitrary distinction between *profiting* from one’s superior knowledge (as low-latency traders do) and *selling* one’s superior knowledge. The latter practice is widely accepted. Millions of exchanges of this kind happen each day when experts sell their knowledge to laymen. By contrast, the former practice might intuitively seem objectionable.

It is possible that there is a morally significant difference here. But I cannot think of how such an argument would proceed. One reason for this is that it seems that, if low-latency traders offered their services to others, they would suddenly also fall in the category of selling their superior knowledge. Quite generally speaking, to come up with a convincing argument that, in a competitive environment, it is morally wrong to be fastest seems no mean feat.

One final thought. Typically, it seems that is not the sellers who complain about LLT, i.e., those people low-latency traders buy shares from. Rather it is the buyers, i.e. ,pension funds like *P*, that low-latency traders sell to.^4^ Indeed, each instance of LLT involves two transactions. First, *T* buys all *C*’s shares from various sellers at $19.83. Second, *T* sells those shares to *P* at $19.84.

The reason I have not engaged with this second transaction in more detail is the following. The one market participant that knows about *P*’s buy order even before *T* does is *P* itself. Thus, if there is a characteristic difference in information levels between *P* and *T*, it is favoring *P*.

Why are buyers like *P* attacking low-latency traders, if not because of an informational advantage the latter have? It must seem *P* is complaining because it would have preferred buying *C*’s shares at $19.83 rather than at $19.84. In other words, it would have preferred if the market only realized that (in light of its own buy order!) *C*’s shares actually are worth $19.84 rather than $19.83 *after* it has bought more shares at $19.83. *P* is basically complaining that the market adjusts too fast for it to profit longer from lower prices. Put differently yet again, it would have preferred if it itself, rather than *T*, knew more than the rest of the market for some time. While understandable, this hardly seems a cogent argument against LLT.

To conclude, not every information asymmetry is morally unsuspicious. They can undermine the legitimacy of an exchange. In three fundamental respects, the case of LLT is not such a problematic case. First, low-latency traders invest money in order to gain an informational advantage in a competitive process which is, in principle, open to everybody (rather than acquiring this advantage in an unjust or *a*just way that calls for rectification). Second, while low-latency traders intentionally continue to know more than others, they do not use this informational advantage to deceive anybody. Third, since LLT relies on public information, it is not a case of insider trading.

What we find, however, is that LLT is suspicious of having a socially wasteful information asymmetry at its center. This reinforces the criticism already surfaced in the context of arbitrage, namely that LLT should be considered socially wasteful in light of the costs and benefits of speed.

## Social Wastefulness, Wrongness, and Speed

The criticism that LLT might be a socially wasteful practice should not make us jump to the conclusion that LLT is morally wrong. However, it calls for a closer analysis of how social wastefulness may influence the permissibility of LLT—and the role speed plays in this. The problem with analyzing this connection is that there is no commonly agreed on definition of what constitutes social wastefulness.

On the one hand, some suggest that low-latency traders are “parasites” in the sense Marx [e.g. (1867) 1909, 505] uses this term; i.e., unneeded middlemen who profit from other people’s work. For their profits are financed by making trades more expensive for others. This thought could form the basis for an argument that LLT is “socially wasteful.” On the other hand, there are Hayekian considerations. If the business model of LLT emerged and is profitable in a market environment where resources tend to end up with those people possessing the highest productive efficiency for them (that is, who are able to maximize outputs relative to the inputs they are given, so that nothing is wasted), that seems to speak against LLT being “socially wasteful.”

Examining whether LLT is socially wasteful against the background of this parasites-versus-profit-earners debate is a route that, even if it suggests itself, we should not take here. This is because what inevitably lurks behind those terms is the much bigger debate whether markets—and the (un)desirable forms of profit-seeking they bring—are a good principle of social organization. That debate is beyond the scope of this essay. And we must be careful that loaded debates between “friends and foes of the market” (Herzog, 2013) do not obscure our analysis of the moral role speed plays when trading at high speeds.

I want to suggest a Lockean route instead. Engaging with Locke’s (1689, 1988) thought we can develop a theory of social wastefulness that is both insightful and fits our purpose here. For Locke not only gives his own definition of wastefulness. He also tells how wastefulness, speed, and wrongness hang together. In the following, I shall develop a liberal Lockean account that, roughly speaking, provides reason to believe that LLT is not socially wasteful (and *a fortiori* not morally wrong) as long as it actually happens fast.

### A Lockean Account

When talking about appropriating resources and wastefulness in the second *Treatise*, Locke’s concern is to prevent that resources get allocated to people who let them spoil.

God has given us all things richly … But how far has he given it us? To enjoy. As much as any one can make use of to any advantage in life *before it spoils*; so much he may … fix a property in. … Whatever is beyond this is more than his share, and belongs to others. Nothing was made by God for man to *spoil*. [Locke (1689) 1988, II, §31; my emphases]

This is Locke’s so called *spoliation proviso*. People are not allowed to appropriate without limits. They only legitimately appropriate what does not then spoil. As Simmons (1992, 283–287) highlights, Locke takes this stance because letting resources spoil is not compatible with God’s intention that we use them for the preservation and comfort of all.

Since their right is to make property … in whatever fair share of the common they choose, I infringe their right by precluding their choice of the goods I waste. One who has wasted resources has thus ‘offended against the common law of nature’ and is ‘liable to be punished’ (II, 37). Waste harms others, even in conditions of relative plenty. (Simmons, 1992, 286)

In the relevant passages (II, §31, §37, §38, §46) Locke talks as if “spoiling” and “using” were opposites and “equates *useful* things with *perishable* things” (Simmons, 1992, 283). This makes his theory of wastefulness somewhat unclear. For there are goods that are useful, but which do not spoil if lying unused (e.g. oil or land). For them, the opposite of use, i.e., non-use, is not the same as spoliation.

Simmons (1992, 285–287) and Waldron (1990, 207–209) discuss different possibilities of how to interpret Locke here, contrasting spoliation with willful destruction, consumption, waste, and non-use. They disagree on the question to what extent non-use qualifies as waste and which kinds of non-use/waste Locke’s proviso rules out. There is, however, an undisputed “core” of the proviso. It clearly forbids to acquire a good to then let it spoil—either negligently or deliberately—where spoiling means a drastic reduction in use-value. Letting goods spoil brings about a situation in which it is as if the goods had never been created, as if they had never been capable of satisfying somebody’s desires, which violates people’s rights.

I propose to make this core of Locke’s proviso the core of our account of social wastefulness. The question then becomes: does LLT violate the core of Locke’s proviso? Three things are important to clarify before addressing that question, to show that we can make use of Locke for our purpose.

First, in which sense would this be a theory of *social* wastefulness? One variant of the social wastefulness-criticism goes like this. The good in question that the low-latency traders acquire is the trading data of agents like *P.* Acquiring these data is costly. The resources spent acquiring it could have been put to better use if allocated to someone else. This is a criticism of LLT being *socially* wasteful, in that waste is about who gets to spend which resources. Seemingly Locke does not address this social side of things with his idea of waste as spoiling goods.

However, I think Locke shows us that there is a different way to conceive of what is social about wastefulness. He shows that we may not only understand wastefulness in the way that a practice that spends resources in one way when there are better ways around is wasteful; i.e., that we may waste resources *relatively* speaking. Following Locke, we may also reasonably hold that a practice which lets resources spoil wastes them; i.e., that we may waste resources *absolutely* speaking.

Locke’s account is what we may call an *absolute* account of social wastefulness. He focuses on what the people do with what they acquired. Without making any comparisons, he simply asks: do they let it spoil? Goods acquired by people who then let them spoil are unjustly acquired. While Locke separates the idea of wasting (causing a drastic reduction in use-value) from ideas of social misallocation, his theory still is a social one. For Locke, goods must not get allocated to people who let them spoil, for they are precluding others from choosing the spoilt goods. Like not allocating goods to people who have a better use for them, this also is a form of social misallocation.

Second, notice that Locke discusses the proviso in the context of how to legitimately transform commonly owned things into private property for the first time. Yet, when setting up an LLT operation, low-latency traders mostly buy things that are already privately owned. As regards this limitation of Locke’s argument, Nozick (1977, 178–179) highlights that Locke’s proviso also casts a shadow on all subsequent transactions. He stresses that whatever we are trying to avoid with a proviso on initial appropriation could likewise result from a series of exchanges. Put differently, Nozick holds that if we want to follow Locke, we *should* also have a Lockean proviso on subsequent exchange of privately owned things. Mildenberger (2020, chap. 6), in turn, argues that we *can* naturally develop such a proviso on exchange and elaborates on what it may look like. According to him, that part of the proviso that reflects Locke’s original non-spoliation condition would be the demand that we must not exchange in a way that would reallocate goods to where they then spoil (2020, 119).

Third, we need to consider in which sense the data acquired may spoil. Physically speaking, the information that *P* wants to buy 500,000 shares of *C* at a certain point in time does not spoil. You can store it digitally without quality loss. However, the data acquired become a worthless piece of information with a use-value of virtually zero in fractions of seconds if not acted on fast. Once the opportunity of profitable trading based on the data is gone, any alternative future use for the kind of data acquired by LLT operations is hard to imagine.

To summarize, we may consider the data acquired a good able to spoil along Lockean lines. So we may hold that its acquisition is subject to the core of the proviso (expanded to the exchange of already privately owned goods). And we can rely on a Locke-inspired absolute account to examine LLT’s social wastefulness.

Now, in a certain sense, LLT is all about not letting the data so costly acquired spoil. Given that LLT is competitive, lots of efforts go into making use of the data before it spoils, i.e., before someone else closes the trade. Low-latency traders pride themselves on being the fastest out there. Their ambition precisely is to let nothing spoil, i.e., to make profits even from the most minuscule price differences existing for the shortest time spans.

In light of this, a Lockean approach provides reason to believe that LLT is not spoliation (and *a fortiori* not morally wrong) as long as it actually happens fast; i.e., with the speed required to use the data before it spoils. This again underscores the moral role speed plays in judging the permissibility of LLT.

### The Limitations of a Liberal Lockean Account

Locke’s reasoning may serve as a qualification to the critique of LLT being a socially wasteful practice and thus wrong. But there are things we might want to object to such a liberal account. To name just the most salient one: that it is too lenient with respect to how few practices it rules out as wrongfully wasteful (cf. e.g. Thompson, 2015, 253–255).

For instance, based on a Lockean account it would be permissible to buy huge quantities of food to produce a kind of food artwork, as long as this creates value (if not the most natural use-value associated with food). In general, the account does not forbid practices that spend even large amounts of resources in creating value of the most various kinds. Locke has a very wide notion of what constitutes legitimate use of resources (“to any advantage in life”; II, §31). As Waldron points out, Locke’s proviso “does not generate any quantitative limit to human possessions” (1990, 209) either. Locke is aware that once there is money, this leniency might contribute to rising inequalities within a society, because money does not spoil [(1689) 1988, II, §46–47]. Yet, the spoliation proviso gives us no reason to limit these effects (Waldron, 1990, 222–225).^5^

One might consider this lenient liberal position untenable—even if there are possible defenses. First, consider that while a Lockean account does not rule out many practices as socially wasteful, it is strict in another sense. Namely, when it comes to the severe consequences for those who violate the proviso. For Locke, violations of the proviso are unjust. If LLT was merely morally wrong, low-latency traders could get away with being blamed for what they do. But Locke argues that violations of the proviso are punishable violations of rights. He is anything but lenient when drawing this straightforward connection from the proviso to matters of justice, i.e., to matters many liberals think are those which are by definition enforceable by the state [e.g. Mill (1861) 1991, chap. 5, para. 13–40; cf. Skorupski (2010), 351–359].

This is particularly significant because the proviso governs both the taking and holding of resources. According to Locke (II, §31, §37–38, §46), we may only take so much as we can reasonably expect to not let spoil; and if any of what we took actually spoils we forfeit our rights over it. In this sense, a Lockean account justifies both pro-active and retroactive regulation and state intervention to avoid spoliation. Only those new LLT operations would be officially sanctioned to lay cables etc. that can show that they can reasonably expect to be still faster. Some liberals might even want to argue that we are justified to pass regulation banning LLT because of how it *risks* to create an injustice (Duff & Marshall, 2015). Also, other than simply waiting for continuously unsuccessful LLT operations to go out of business, on Lockean grounds we could argue for their dispossession. For they acquired what is beyond their share and “belongs to others” [Locke (1689) 1988, II, §31]. As Harding notes, we can “of course quibble about the nature of the claim these ‘others’ may have, and whether this amounts to outright transfer of ownership” (2020, 301). Still, on Lockean grounds acquiring what then spoils is retrospectively unjust and the too slow traders guilty of a transgression of natural law—which justifies a removal of exclusive rights (Simmons, 1992, 287).

To summarize, a Lockean account has a narrow domain, and might be criticized to be lenient in light of this. But it is a forceful account if the conditions for spoliation are met.

The second defense is that cogent less lenient accounts of social wastefulness are more difficult to come by than one might *prima facie* think. Consider *relative* accounts of social wastefulness. As briefly introduced above, relative accounts define waste in terms of how resources could have been put to better use elsewhere. They are a manifest option, as it seems to be a common intuition that LLT, even if not guilty of spoliation, is comparatively wasteful—an inefficient use of resources—and wrong for that reason.

The problem with relative accounts is that they are highly susceptible either to being over-demanding or to offering no guidance on what is *wrongfully* wasteful. Thompson, putting forward such an account, explicitly states that it is waste “whenever some thing, or some person … is unable to bring forth into the world the maximum of its abilities and potentialities” (2015, 255–256). This condition will almost always be met, i.e., there will virtually always be waste. Consequently, Thompson openly admits his position of morally criticizing practices that spend resources inefficiently as being wrongfully wasteful is “a demanding ethic” that is not “is always capable of being satisfied to its fullest extent” (2015, 269). For we may always do so, unless we are looking at the most efficient practice.

In order to better illustrate the risk of either over-demandingness or offering insufficient guidance that relative accounts introduce, an analogy to *effective altruism* might help (e.g. MacAskill, 2015; Singer, 2015). For effective altruists face a structurally similar problem. They want to allocate the money at their disposal in such a way that it maximizes well-being. They ask themselves whether the dollar they just allocated on buying ice cream was not inefficiently spent, in the sense that it could increase well-being more if it was allocated differently. It almost always can, which makes effective altruism notoriously prone to the critique of being over-demanding (cf. Chappell, 2009).

Consider Singer (2015, 28–31) telling the story of Julia, an enthusiastic effective altruist struggling with the fact that her commitment to effective altruism is at odds with her occasionally having an ice cream.

When shopping, she would constantly ask herself, ‘Do I need this ice cream as much as a woman living in poverty elsewhere in the world needs to get her child vaccinated?’ That made grocery shopping a maddening experience, so she and ... [her husband] made a decision about what they would give away over the next 6 months [– namely, 50 percent of their income –] and then drew up a budget based on what was left. Within that budget, they regarded the money as theirs, to spend on themselves. Now Julia doesn’t scrimp on ice cream because, as she [says] …, ‘Ice cream is really important to my happiness.’ (Singer, 2015, 29–30)

It is easy to see how big a relief Julia’s solution of drawing up a budget and spending note more than 50% of her income on donations is. Singer (2015, 29–31) endorses this way of thinking, i.e., that we are not doing something wrong if we spend money on ourselves within a certain budget, as a good practical solution.

Unfortunately, such “threshold”-ideas come at a cost. We may define social wastefulness relatively speaking. And we may separate this definition from wrongfulness, arguing that only some instances of social wastefulness are wrong. Namely, those that surpass a certain threshold; that are, as it were, too wasteful. This effectively solves the issue of over-demandingness (just as the 50% budget solves Julia’s problem). But it comes with the burden of having to justify that threshold. Since the threshold needs an anti-maximization component to work effectively, it cannot be based on the same logic we used in defining social wastefulness relatively. We have to introduce considerations that are not directly related to our definition of waste.

The risk of introducing theoretical inconsistencies this way is real. At the very least we may conclude that, unless we opt for the demanding variant that all wastefulness is wrongful, relative accounts offer no guidance out of themselves of what constitutes wrongful social wastefulness. (Just as the 50% budget is not derived from within effective altruism.) By contrast, a Lockean absolute account does not raise these worries. Every instance of spoliation is wrongful, without this being over-demanding.

That relative accounts offer no guidance out of themselves might be seen as a minor shortcoming if there were an established independent account of how to measure wastefulness and compare it across widely differing practices. Alas, there is not. Supposedly, in order to do so, we would have to come up with some scale against which to measure wastefulness in terms of some highly abstract value (like, maybe, disutility). On this scale, we would then be able to answer questions like “How wasteful is LLT?” and “How wasteful is practice *p*?” by giving a number for each. I doubt this can be done in a cogent way.^6^

Here is a third defense. I suspect that some may intuitively find the liberal position too lenient for reasons that are not strictly speaking related to wastefulness. Notably, some may be inclined to say that we waste resources when pursuing the wrong end, even if we pursue it efficiently. They may say, for instance, that efficiently pursuing a career in theater is “a waste,” if the person in question also could have made a high-paying career in business.^7^

But while we may speak of waste in these circumstances, these are first of all questions about which ends we deem worthy of pursuit. We wonder which of many possible ends we and others should pursue—with relative efficiency or absolute non-spoliation being tacitly assumed or considered independent *desiderata*. Tellingly, Thompson (2015, 258), who holds that efficiently pursuing the wrong ends is a kind of wastefulness, refers to these cases as mis-*directions* of resources rather than as under-*uses*.

Locke’s answer to the question of which ends are worthy of pursuit is a classical liberal one. We may use what we do not let spoil “to any advantage in life” [Locke (1689) 1988, II, §31] we choose. This makes his account lenient in this regard. It allows for pursuing LLT based on classical liberal reasons. Yet, a non-Lockean absolute account of social wastefulness would be compatible with alternative theories about which ends we should pursue. This includes theories that would make such an account less lenient (potentially even over-demanding), like perfectionist ones. Again, Thompson may serve as an example. He holds that mass entertainment by means of professional sports or Hollywood movies is not a “socially valid” (2015, 256) end, even if pursued in neither relatively nor absolutely wasteful ways.

That compatibility of absolute accounts strikes me as a positive feature from a theoretical perspective. Discussing alternative theories about which ends are worthy of pursuit is beyond the scope of this essay. But these considerations show how we could make a non-Lockean absolute account less lenient overall in matters that are not about social wastefulness strictly speaking.

To conclude, relying on Locke is a decidedly liberal way to qualify overblown claims about trading at high speeds being socially wasteful. Relying on Locke, we can offer a consistent and well-justified absolute account of social wastefulness that is strict in some ways, lenient in other ways, and flexible in yet others. Importantly, it provides answers that bypass discussing things that are beside the point in our context, e.g. the moral status of profits and markets, or which ends are worthy of pursuit. Instead, the account highlights the moral role speed plays. What an argument based on Locke’s spoliation proviso gives us is a reason to believe that LLT is not socially wasteful (and *a fortiori* not morally wrong) as long as it actually happens fast.

## Systemic Risk

As regards the question of whether speed as such on markets is morally neutral, the *systemic risk* posed by LLT is particularly salient. If speed as such is wrong, then maybe that is because millions of fast transactions might quickly lead to disaster. “Flash crashes” like that of May 6, 2010 or February 5, 2o18 in which the financial markets fall at a hitherto unseen pace support this worry. Speed is a necessary condition for the potential harm caused by such crashes.

As Moggia points out, “systemic risk in finance is mostly created collectively, while the individual contributions to it, considered in isolation, seem to be harmless (the problem of unstructured collective harm)” (2021, 461). In our context, the worry is that the unstructured collective harm (of a stock market crash) might ensue even though the individual transactions performed by low-latency traders might be morally speaking unsuspicious. Another worry is that, in cases of systemic risk, the unstructured collective harm typically is an *externality* to the seemingly harmless transactions (cf. Linarelli, 2017, 331). That is to say, it is not only the low-latency traders who lose money in the crash, but also many “bystanders.” Finally, what characterizes the harm done by systemic risk according to Linarelli (2017) is that the system unravels quickly once certain thresholds or tipping points are crossed. Roth’s (2015, 82–86) description of the events of May 6, 2010 highlights that all of these elements characterizing systemic risk were present in the flash crash that day.

If LLT imposes the externality of systemic risk on financial markets, we might be inclined to consider LLT wrong because of this. However, there is no straightforward connection between the premise that certain transactions feature an externality that imposes a risk of harm and the conclusion that these transactions are wrong.

Examining externalities in general, Mildenberger (2018) argues that, at least on liberal grounds, such a conclusion is typically blocked, even if the premise is true. This is because one typically cannot show that exchanges featuring externalities *wrongfully* risk a harm. This would be required for the argument to go through. But in order to argue that exchanges featuring externalities indeed wrongfully risk a harm, one has to rely on controversial ideas like *associative duties* of sellers toward buyers (cf. Dworkin, 1986, 195–216) or on stretching an analogy with the legal doctrine of *aiding and abetting* crimes (cf. Duff, 1990, 168–174). While this is an option, such an argument would ultimately require us to subscribe to particularly demanding variants of liberalism, like Raz’s (1986) perfectionist liberalism (Mildenberger, 2018, 2115–2119).

These considerations highlight that it is by no means easy to make a convincing analytical case that LLT is wrong because it imposes the externality of systemic risk. But let us assume, for the sake of the argument, that we can come up with a cogent argument connecting premise and conclusion. In this case, we would still be left with the problem of showing that LLT indeed imposes the externality of systemic risk on financial markets. For while there is some evidence that this premise is true, the case is not clear-cut.

Here are two considerations which speak against LLT imposing a systemic risk. First, note that the transactions effected by low-latency traders do not resemble the transactions which have contributed to the 2008 financial crisis. Arguably, risky transactions featuring not *information* asymmetries but *expectation* asymmetries played a significant role in causing that crisis (Claassen, 2015; de Bruin, 2018; Moggia, 2021). In cases of expectation asymmetries, each party individually expects something that would not be rational to expect if there was shared information. Transactions involving expectation asymmetries are similar to a duel which each is willing to fight only because each believes he is much more likely to win; but which no one would be willing to fight if each had a 50 percent chance of winning, which is the probability that both parties would rationally have, if they had shared information. Many transactions involving future-oriented financial products seem to have a similar structure with respect to expectation and risk. And there are reasons to believe that the systemic risk created by such transactions—namely, the risk of a lot of expectation asymmetries unraveling quickly and at once—was one of the main causes for the financial crisis of 2008.

By contrast, the transactions effected by low-latency traders do not involve expectation risk. LLT relies on an information asymmetry that lets you buy things now of which you know there is a demand for a split-second later. Thus, LLT at least does not build up the same systemic risk responsible for one of the worst stock market crashes in history.

**Fig. 1:**
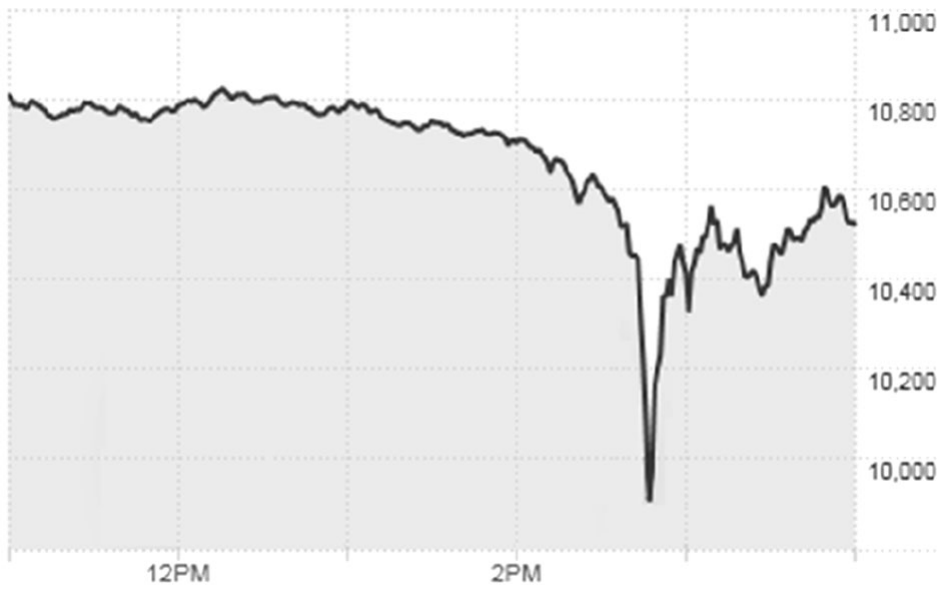
*Dow Jones Industrial Average* intraday chart for May 6, 2010 (CNN, 2010)

Second, it is noteworthy just how flash crashes look. Figure 1 shows the intraday chart from 11 am to 4 pm of the *Dow Jones Industrial Average* on the day of the flash crash of May 6, 2010.

Looking at Fig. 1, it is easy to see that what characterizes flash crashes is not only that stock prices fall at unprecedented speeds. They also bounce back rather quickly. The *Dow Jones Industrial Average* fell about 9% in mere minutes only to recover most of its losses after about half an hour. This holds true for other flash crashes as well. As soon as one leaves the intraday view of stock indices behind, they visually disappear. This stands in stark contrast to “regular” stock market crashes. Here, stock prices are declining less rapidly but for a longer period of time. Likewise, it takes a lot longer for markets to recover.

This is not to say flash crashes are no real risk at all, or that they can never produce significant financial harm. But judging from a systemic risk perspective, “regular” stock market crashes like the 2008 financial crisis, which led to the Great Recession and almost brought the global financial system down, are considerably more troubling. In all flash crashes up to date, the system did not even come close to breaking down but rebounded within minutes. This is because people are aware that the reason for flash crashes is a malfunctioning of trading algorithms, rather than genuinely bad news as regards the global economy.

There are two other *prima facie* promising lines of arguments one might have in mind to show how LLT creates systemic risk—but neither withstands scrutiny. First, low-latency traders themselves, if they want to highlight that their business model also is socially useful, often claim that they are *market makers*. A market maker is a third party on an exchange that stands between buyers and sellers, connecting them. There are some market makers that are officially licensed by the exchanges they operate at. They have the contractual duty to always be willing to buy and sell a certain predetermined quantity of the securities they are licensed for. They thus ensure the liquidity of the market.

Factually speaking, low-latency traders often act just like market makers. But this stance should not be confused with them actually being officially licensed market makers. For they have no contract with the exchange they are operating at. They may choose to act as a market maker at their leisure, without having to respond to any moral obligation in this respect.

Consequently, if an exchange chose to exclusively rely on low-latency traders as market makers, and a situation came to pass in which all low-latency traders chose to cease operations, the market might break down. Willing sellers might be unable to find buyers and vice versa. Thus, relying on LLT alone to do the market making creates systemic risk.

Yet, if this scenario ever became reality, it would be curious to blame low-latency traders for this. It is the duty of the people operating the exchange to ensure liquidity. By contrast, lacking a contract with the exchange, low-latency traders strictly have no duty to make the market.

A second, ultimately unconvincing line of argument goes as follows. LLT profits from volatile markets. If many people are frantically trying to buy and sell, and bid-ask spreads are high, emotionless trading algorithms can make the most money. Thus, there is an incentive for low-latency traders to generate volatile conditions that pose a systemic risk. But just because LLT profits from volatility does not mean that low-latency traders actively create it. For one thing, because it is illegal to manipulate the market in this way. This means that we are at most we are dealing with an instance of some low-latency traders engaging in a *malum prohibitum*, i.e., a kind of conduct whose wrongfulness depends essentially on its illegality (Duff, 2007, chap. 4.4). But this does not make LLT a *malum in se*.

On a curious note: the systemic risk one suspects LLT to create might rather lie elsewhere. Consider that electronic trading—which forms the basis of all LLT—only became standard after the Black Monday of October 19, 1987. On that day human traders simply stopped to pick up their phones, since they did not want to handle even more clients wanting to sell their stocks as quickly as possible. In the aftermath of the crash, exchanges were looking into alternative ways of trading to eliminate the human factor (and the systemic risk created by it). They judged that electronic trading effected by machines was the safer way to go (Moser & Wunderer, 2020).

## Implications for Other HFT-Techniques

Beyond what has been said about speed’s moral role for LLT, the findings have implications for the wider HFT-industry. We should be careful to distinguish, however, between those techniques that are all about being fastest, and those which simply can be executed better (or more often) if the trader is comparably faster. Sorting the HFT-techniques mentioned by Aldridge (2013, chaps. 8–12) and the list of purportedly malicious HFT-techniques compiled by the *Commodity Futures Trading Commission*’s subcommittee on HFT (CFTC, 2021) along these lines gives us the following picture. Techniques relying on *large-to-small information spillovers*, *futures arbitrage*, *latency arbitrage*, and *quote stuffing* fall into the former camp. *Statistical arbitrage strategies, directional strategies*, *informed market making, spread scalping*, *rebate capture*, *quote matching*, *spoofing*, and *pinging/sniping/sniffing/phishing* techniques fall into the latter camp. They have a speed component, but also feature other aspects.

Consider directional trading around events. It sure helps if one is the trader who can react fastest to new macroeconomic data being publicly released. There is a straightforward analogy to LLT here. However, to practice successful directional trading one also needs an informed estimation about how the news will affect the market. For example, whether an inflation rate of, say, 2.1% will lead to rising or falling stock prices.

For directional strategies, it is a combination of fast reaction speeds and informed predictions that is needed for success. For LLT, no prediction whatsoever is needed—and thus no resources need to be allocated to making good predictions. *P*’s buy order *ceteris paribus* will raise the market price as all buy orders do. Because something beyond speed is needed for strategies falling in the latter camp, in the following I shall have to say more on the implications of our findings as regards strategies that are all about being fastest.

Techniques relying on *large-to-small information spillovers* or *futures arbitrage* both rely on the idea that some securities react faster to changes than others. The fastest trader can then profit from bringing the news from the faster-reacting market to the slower-reacting market (Aldridge, 2013, chap. 8). For instance, “small” stocks, i.e., stocks of firms with a relatively limited market capitalization, are known to react slower than “large” stocks. Likewise, “futures markets have been shown to adjust more quickly than spot markets” (Aldridge, 2013, 143).

Both techniques fundamentally are arbitrage. They exhibit the features underlying arbitrage’s social usefulness as outlined by Hayek. Notably, they connect different marketplaces. The same holds for *latency arbitrage*, where high-frequency traders sell a stock “in the market where the stock is temporarily overpriced, while simultaneously buying it where the stock trades too cheaply” (Aldridge, 2013, 197).

Thus, these techniques are like LLT in that only the fastest can profitably execute the corresponding trades, as the price differences disappear almost instantaneously. But they are unlike LLT in that they clearly contribute to equilibrate market prices in previously divergent markets. They help to bring actual markets closer to ideal theoretical market conditions in which we should only see one price across markets (the “law of one price”). Traders practicing these techniques have been found, for example, to narrow bid-ask spreads, increase liquidity, and reduce intraday transitory pricing errors and volatility (Litzenberger et al., 2012). Thus, the contrast between the benefits and costs of arbitraging at high speeds is reduced for these techniques.

The situation is quite different for *quote stuffing*. Quote stuffing refers to the practice of rapidly entering and withdrawing orders to flood the market, making orientation for other traders more difficult (Jain & Jordan, 2017, 378). “The alleged intent is not to trade, but to slow down other traders whose computers are slowed down by all the message traffic” (Angel & McCabe, 2013). After all, you cannot only be faster than others by having a lower latency yourself, but also by increasing other traders’ latency.

Quote stuffing has been under legal scrutiny, but it is not officially deemed illegal. Although it would be comparatively easy to end this practice by requiring a minimum time for an order to be held open, such regulation is not in place at most major stock exchanges (Holzer & Philbin, 2010; Moser & Wunderer, 2020). We can now see more clearly, though, why it might nevertheless be considered morally wrong.

If a trader succeeds in quote stuffing, this is problematic precisely in those cases in which speed has been argued to be morally desirable, e.g. as it protects LLT from being wasteful. One can imagine a scenario in which successful quote stuffing by stuffer *S* prevents low-latency trader *T* from closing a deal. In this case, *T*’s efforts to acquire an informational advantage were in vain.

Arguably, quote stuffing is best understood as a competitive practice among low-latency traders, not intended to affect the market at large. Orders are placed and withdrawn so quickly that no high-latency trader could possibly fill them. Thus, if *S*, on top of preventing *T* from closing the deal, closes the deal in his stead, one might argue that no harm other than a “market harm” (Thomson, 1986, 160) has been done.^8^

This picture might be too positive, though. Aldridge argues that quote stuffing cannot possibly work in the way described and actually belongs to the “purported” strategies of high-frequency traders. This is because,

as any network engineer will confirm, an individual network account cannot selectively slow down network communication for some participants, while still receiving high-speed access to the trading venue. When a matching engine gets clogged with orders and cancelations, it is equally clogged for all market participants, irrespective of who caused the problem. Obviously, such network-clogging exercises do little for traders equipped with fast technology; if anything, network clogging only voids the benefits of fast technology. (Aldridge, 2013, 202)

If Aldridge is correct, then quote stuffing is a prime example for a trading technique that exhibits those kinds of moral flaws commonly attributed to LLT. Namely, to be inherently wasteful. Also, a Lockean defense of this practice would be hard to come by. The very intention with which the technique is performed is to create spoliation.

Quote stuffing thus creates moral problems tied to speed, as it messes with the good effects speed has in other respects. Empirically speaking, it has been found to decrease stocks’ liquidity, lead to higher trading costs, and incite increased short-term volatility (Egginton et al., 2016). Quote stuffers may also choose to target entire market feeds rather than individual securities. This is because, with a large number of orders placed, orders will pile up in the backlog of the market data feed, thereby increasing the latency of the entire feed. (Diaz & Theodoulidis, 2012). If they do so, they might foil many of the efforts of the wider HFT-industry.

Our findings also yield some implications for the speed component of those HFT-techniques that are not all about speed. There is no difference in kind between, for instance, futures arbitrage and the speed component of directional trading strategies around events. Both techniques help to equilibrate markets quickly. By contrast, if there are intentional efforts to counteract the speed component of other HFT-techniques, our findings suggest that they are, *pro tanto* and *prima facie*, to be judged negatively.

## Conclusion

LLT is not that kind of arbitrage that Hayek defends as socially useful. And while not in principle impermissible because of the information asymmetries it features, Steiner’s account helps to highlight how an overall judgment of its legitimacy may be negatively affected by the costs associated with reaching the high speeds required. Finally, past flash crashes forebode how things might go horribly wrong in future and highlight the kind of risk LLT exposes financial markets to because of the speed with which it proceeds. This is what arguably is wrong with high-frequency trading in the form of LLT.

A critique of LLT along these lines needs to be a qualified in at least three respects. First, based on Lockean thinking, we may come up with a fitting liberal argument as to why LLT, in spite of its high costs and seemingly small benefits, is not socially wasteful after all as long as it happens fast. Second, it is analytically hard to argue and empirically difficult to show that LLT even has true destructive potential in the form of flash crashes. Third, it is important to qualify the critique by limiting its domain. What holds true for LLT does not necessarily hold true for other HFT-techniques that are all about speed. Futures arbitrage, for instance, does exhibit the social benefits of arbitrage to a greater extent. While the case of LLT is suitable to highlight how speed morally matters in the context of HFT, we should not base a general verdict about HFT on the case of LLT alone.

All things considered that LLT is situated somewhere between strict permissibility and strict impermissibility. What we do see more clearly now is that speed in financial markets is not morally neutral.

## Footnotes

1 If I speak of more “accurate” pricing here, what I have in mind is the mainstream economic idea that prices are more accurate the more information they incorporate (e.g. Varian 2006). Any *additional* informational connection thus increases price accuracy.

2 LLT is a competitive industry, however, so that low-latency traders do not make a *fully* risk-free profit when buying shares at exchanges *F*, *G*, and *H* to sell them to *P*. They can be sure that, eventually, *P*’s buy order will reach these exchanges. There is no risk here. But they cannot be sure that they will have been the fastest low-latency traders. Another trader might have been even faster, taking away the profit they thought to be secure.

3 Cooper et al. (2016) present an insightful argument in this respect, namely that LLT *should not* be considered deceptive. This is because, according to them, the threshold for considering a certain practice deceptive has to be particularly high in financial markets “where almost any regulation, other than the most basic, tends to create a moral hazard and increase information asymmetry” (2016, 1). That is to say, they basically argue that we have to be careful not to worsen the issue of information asymmetries being present by establishing stricter regulation on deception.

4 A prominent example is the city of Providence, which filed a lawsuit against major exchanges and banks because of suffering from LLT (Martens, 2014).

5 One thing it does rule out—because of how it is motivated—is “the case in which a man accumulates resources purely to beggar his neighbors, to diminish their ability to satisfy their needs” (Waldron, 1990, 208–209). For this would be contrary to God’s plan to give the earth to mankind for the preservation of all. NB: Locke’s overall account also comprises the “enough and as good” proviso which creates limits of its own (cf. Simmons, 1992, 282–284). Arguably, it rules out cases like the food artwork.

6 Examining less lenient absolute account of social wastefulness, we run into analogous problems. To satisfy those criticizing Locke’s account for being too lenient, it seems we would have to come up with an account that not only considers non-use and spoliation as wrongfully wasteful uses of resources, but even some productive uses. The question that then arises—how productive is productive enough?—mirrors the question of how wasteful is too wasteful.

7 Effective altruists subscribing to the idea of “earning to give” would be one example (MacAskill, 2015, chap. 9).

8 Thomson argues that wherever there is competition in markets, there will be winners and losers. But as long as the rules of competition are fair, the harm incurred by the losers does not qualify as a wrongful harm.
